# Whispering Gallery Mode Thermometry

**DOI:** 10.3390/s16111814

**Published:** 2016-10-29

**Authors:** Simone Corbellini, Chiara Ramella, Lili Yu, Marco Pirola, Vito Fernicola

**Affiliations:** 1 Department of Electronics and Telecommunications, Politecnico di Torino, Corso Duca degli Abruzzi 24, Torino 10129, Italy; simone.corbellini@polito.it (S.C.); chiara.ramella@polito.it (C.R.); marco.pirola@polito.it (M.P.); 2 College of Electrical Engineering and Control Science, Nanjing Tech University, Nanjing 211816, China; 3 Istituto Nazionale di Ricerca Metrologica, Strada delle Cacce 91, Torino 10135, Italy

**Keywords:** whispering gallery mode resonators, temperature sensors, high-accuracy thermometry, microwave resonance, sapphire-based dielectric thermometry

## Abstract

This paper presents a state-of-the-art whispering gallery mode (WGM) thermometer system, which could replace platinum resistance thermometers currently used in many industrial applications, thus overcoming some of their well-known limitations and their potential for providing lower measurement uncertainty. The temperature-sensing element is a sapphire-crystal-based whispering gallery mode resonator with the main resonant modes between 10 GHz and 20 GHz. In particular, it was found that the WGM around 13.6 GHz maximizes measurement performance, affording sub-millikelvin resolution and temperature stability of better than 1 mK at 0 °C. The thermometer system was made portable and low-cost by developing an ad hoc interrogation system (hardware and software) able to achieve an accuracy in the order of a few parts in 10^9^ in the determination of resonance frequencies. Herein we report the experimental assessment of the measurement stability, repeatability and resolution, and the calibration of the thermometer in the temperature range from −74 °C to 85 °C. The combined standard uncertainty for a single temperature calibration point is found to be within 5 mK (i.e., comparable with state-of-the-art for industrial thermometry), and is mainly due to the employed calibration setup. The uncertainty contribution of the WGM thermometer alone is within a millikelvin.

## 1. Introduction

Whispering gallery mode (WGM) resonators are, at present, of great interest as high-sensitivity sensors in many application fields [1]. A whispering gallery resonator is basically a solid dielectric (either crystalline or amorphous) with a symmetric shape with respect to a rotational axis (e.g., a cylinder, a sphere, a rod, a disk, etc.). Whispering gallery modes are resonant modes of high-order azimuthal number that have most of the energy concentrated on the dielectric’s surface and can be thus described with a propagation mechanism analogous to optical total internal reflection that occurs at the dielectric–air interface. WGMs are very high-Q modes, thus offering good suppression of spurious modes [2].

The study of WGMs started almost a century ago by Lord Rayleigh, who investigated the propagation of sound over a curved gallery surface [3,4]. Later on, in 1909, Debye derived equations for the resonant eigen-frequencies of free dielectric and spheres, taking WGMs into account [5]. Since the middle 1970s, microwave WGM resonators have been investigated as the basis to implement high-stability, low-phase-noise microwave oscillators [6]. Sapphire is mostly used as a dielectric to implement microwave WGM resonators, thanks to its great advantages in terms of low dielectric loss and good chemical stability, thermal conductivity, and light transmission. Optical WGM microresonators have also been widely developed in the photonic field, yielding to the implementation of micro-oscillators and highly selective optical filters [7].

In the typical structure adopted for microwave WGM oscillators, the sapphire resonator is suspended within a vacuum-pumped resonant cavity. The latter has the function of optimizing electromagnetic (EM) coupling and protecting the dielectric’s surface from any contamination that may cause a change in the resonance frequency, due to the superficial nature of WGM wave propagation. However, sapphire-based microwave WGM resonators show a high sensitivity to temperature, due to the large permittivity change with temperature. For this reason, temperature compensation techniques were developed to achieve state-of-the-art WGM oscillator performance at cryogenic temperatures.

More recently, by completely reversing the point of view, it was found that the superficial nature and the thermal sensitivity, together with the very high Qs, of whispering gallery modes, can be exploited for implementing high-resolution WGM-based sensors. Nowadays, it is possible to find plenty of literature on WGM sensors, of which a detailed review can be found in [1]. Applications include temperature, pressure, force and displacement sensing, EM-field strength measurement, humidity and gas evaluation, nanoparticle detection, and biosensing.

The present work focuses on thermometry applications of sapphire-based microwave WGM resonators. In 2007, the first whispering gallery mode thermometer (WGMT) was proposed by National Institute of Standards and Technology (NIST), achieving uncertainty around 10 mK [8]. In 2012, two more prototypes were developed and characterized at Istituto Nazionale di Ricerca Metrologica (INRIM), confirming the very good performance achievable by WGMTs [9,10,11]. Results showed that WGMTs can effectively replace standard platinum resistance thermometers (PRTs) in industrial applications, bringing the advantages of a higher mechanical robustness and a relatively lower cost. The cost of a sapphire-based WGMT is comparable with the cost of a good PRT (i.e., in the range of 1 to 2 k€), however, it could significantly decrease in the case of larger-scale production.

The two main issues that limited a widespread use of WGMTs with respect to PRTs are the physical dimensions of the metallic cavity required by WGMTs (at present several centimeters, but potentially reducible) and the cumbersome and expensive laboratory equipment employed for interrogation and data acquisition, typically consisting in a vector network analyzer (VNA) and a frequency reference standard. Those instruments are indeed available in a wide range of sizes and prices and, consequently, of accuracies. However, the high Qs of WGM resonators pose highly demanding requirements on the instrumentation accuracy, thus limiting the possible choices only to expensive and bulky high-performance models. Since in a WGMT the frequency range of interest is known in advance, a low-cost, portable acquisition system—affording limited functionalities with respect to a VNA, yet with a high-accuracy—has been designed and built by the authors [12,13,14,15]. With and overall cost below 7 k€, the developed instrument represents a key enabling element for the widespread use of WGMTs in place of PRTs, which instead would require accurate thermometry bridges whose cost is in the range of 20–40 k€.

In this work, the design of the WGMT system and its experimental characterization are reported, summarizing and integrating the information available in literature [9,10,11,12] and presenting novel calibration results in the temperature range 0–20 °C. The high resolution and low measurement uncertainty obtained in such a narrow temperature range show the potential of the WGMT as interpolating thermometer for the dissemination of the thermodynamic temperature scale, and as a powerful tool for investigating the characteristics of the present interpolating standard PRTs.

## 2. Materials and Methods

The developed whispering gallery mode thermometer system is composed of two parts: a sensing element (i.e., the WGM resonator that transduces a temperature variation into a resonance frequency shift) and a sensor acquisition system. The latter consists in an ad hoc portable, low-cost, vector transmission (S_21_) analyzer equipped with its own accurate frequency reference, and some PC software to control the developed instrument, drive the laboratory testing and calibration setup, identify the resonance frequency from the gathered S_21_ spectrum, and perform thermometer calibration and other performance analyses.

### 2.1. WGM Sensor

The developed sensor is based on a monocrystalline sapphire (Al_2_O_3_, purity 99.996%) WGM resonator. The choice of material was dictated by the good thermal properties of sapphire, primarily the high thermal conductivity, which ensures a uniform temperature distribution in the crystal. The low tangent loss of sapphire (less than 10^−6^) [6] ensures low EM energy dissipation and thus very high resonance quality factors, in excess of 10^4^ [8].

Most important, the relative resonant frequency shift due to the thermal sapphire thermal expansion, in the order of 10^−6^/K, is comparatively smaller than that related to its permittivity change with the temperature, in the order of 10^−5^/K. Therefore, a good approximation of the resonant frequency temperature sensitivity can be written considering only the terms related to permittivity variation, as

(1) $$\frac{1}{f_{0}}\frac{\partial f_{0}}{\partial T}=\frac{1}{f}\left(\frac{\partial f_{0}}{\partial \varepsilon_{┴}}\frac{\partial \varepsilon_{┴}}{\partial T}+\frac{\partial f_{0}}{\partial \varepsilon_{║}}\frac{\partial \varepsilon_{\left|\right|}}{\partial T}\right)$$

where $\varepsilon_{┴}$ and $\varepsilon_{║}$ are the dielectric permittivities in the radial and axial directions, respectively. The strong permittivity dependence on temperature is the source of the high sensitivity that characterizes WGM-based thermometers.

Two dielectric resonator shapes were investigated: cylindrical and spherical. Cylindrical HEMEX sapphire crystals, 12 mm outer diameter and 12 mm height, were purchased from Crystal Systems Inc., while spherical sapphire crystals, with the same outer diameter, were obtained by Rubis-Précis. A cylindrical copper cavity has been designed to optimize EM field coupling and sensor shielding. According to the results published by NIST [8], the best matching is expected to occur when the cavity inner dimensions are twice the resonator size. Thus, the employed cavity has 24 mm inner diameter and 24 mm height. The cavity comprises a cylinder copper body, two copper disks acting as top and bottom sealing caps, and two microwave antennas (SubMiniature version A (SMA) coaxial cables terminating with a short piece of uncoated conductor) tin-brazed to the top disk. The cavity is gold-plated in order to increase surface electrical conductivity and improve electrical contact between the body and the disks. Vacuum sealing is ensured by two Viton^®^ O-rings. The cavity is vacuum-pumped to avoid any pressure-dependent effect on the resonance frequency and/or condensation of water vapor when the thermometer is operated below the ambient dew point temperature. The WGM resonator is suspended at the center of the cavity by means of a 2 mm diameter bolt and two nuts and washers. Both metallic (brass) and plastic (nylon) nuts and washers were tested, showing no difference in the results. In fact, by the radial surface nature of the chosen whispering gallery modes, perturbations along the *z*-axis (aligned with the optical *c*-axis within ±1°) are expected to cause negligible effects on the resonant frequency. On the contrary, the vertical position of the sapphire (i.e., its distance from the antennas) sensibly affects signal coupling. Therefore, a careful preliminary optimization is required to obtain the highest signal strength.

**Figure 1:**
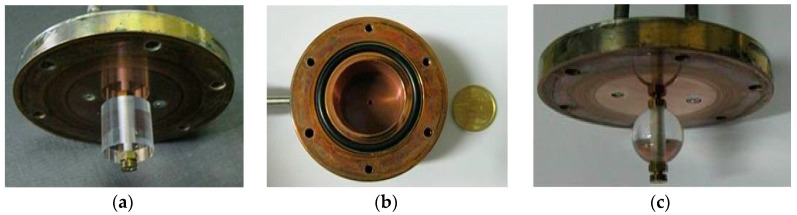
Pictures of the INRIM whispering gallery mode (WGM) resonators: (**a**) top disk with suspended cylindrical sapphire; (**b**) copper resonator body; (**c**) top disk with suspended spherical sapphire.

Figure 1 shows some pictures of the WGM resonators and of the copper cavity.

Finite element method (FEM) analysis was carried out in order to locate the resonant modes of the two resonators. Table 1 and Table 2 report the simulated resonance frequencies ($f_{0}$) and associated quality factors (Qs) of the cylindrical and spherical resonators, respectively. For both resonators, the modes characterized by azimuthal number m of 3, 4, and 5 have been considered, which span over the 10–20 GHz range. This frequency range is sufficiently higher than that of the strongest modes of the external cavity, yet sufficiently low that it does not pose too demanding requirements on the microwave instrumentation employed for resonance measurement. Results indicate that the theoretical (lossless) *Q* of the WGMs increases with frequency (i.e., with m) with the spherical sapphire having slightly better *Q*s for the same m thanks to the relatively higher $f_{0}$.

Note that, for simplicity, simulations were performed assuming the copper surfaces as perfect electric conductors and the sapphire as lossless. Thereby, an experimental Q much lower than the calculated one, potentially in the order of 10^4^, is expected, mainly due to the sapphire dielectric losses.

**Figure 2:**
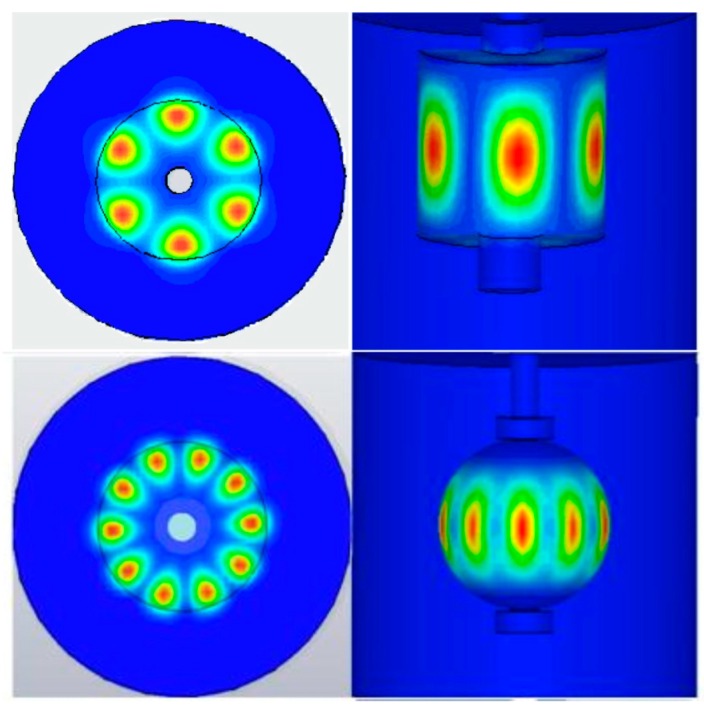
Examples of simulated electric field distribution within the resonators. Upper section: cylindrical resonator, mode m=3. Lower section: spherical resonator, mode m=5.

Two examples of simulated electric energy distribution within the sapphires are given in Figure 2. The field distribution, concentrated along the equatorial circle, clearly indicates that the impact of the central hole, of the assembling means, and of the assumed boundary conditions is negligible. Another interesting result that can be appreciated from Figure 2 is that the azimuthal components are related mostly with the diameter of the sapphire, while the height and geometry are less influent.

Preliminary investigations showed that both resonator sensors are capable of working as thermometer systems and performing temperature measurements with uncertainty comparable to that of industrial PRTs. Sensor interchangeability and reproducibility also are comparable with those of industry-standard PRTs [9,10]. In particular, it is worth noting that the latter mainly comes from variability in mechanical parameters, such as the torque applied to the holding screw and the distance between antennas and sapphire, which is unavoidable in the manual assembling process.

The spherical resonator was adopted for the final thermometer system since it proved to be more accurate and stable. In particular, the whispering gallery mode with m=3 (around 13.6 GHz) was chosen, since it demonstrated, under measurements, the best quality factor. The lower quality factors observed for the higher modes, in contrast with the results of Table 1 and Table 2, is due to dielectric and conductive losses, both neglected in simulation and increasing with frequency, and to the interaction between whispering gallery and cavity modes.

### 2.2. Acquisition System

The most practical way to characterize a microwave resonance is by measuring the scattering parameter S_21_ (transmission between a transmitting and a receiving antenna) of the resonator. As demonstrated in [12], to achieve an accurate resonance measurement, both magnitude and phase of the S_21_ parameter are necessary to account for phase rotations along the cables, thus a vector instrument is required.

Due to the very high quality factors of whispering gallery modes, the instrument accuracy and frequency resolution required to accurately identify such resonances are very high. Therefore, the WGM sensor acquisition system usually needs a high-performance vector network analyzer (VNA) in combination with a highly stable frequency reference. This setup can be efficiently adopted in laboratory experiments. However, to exploit WGM resonators for practical temperature measurements, a portable and low-cost acquisition system must be developed, to form, in conjunction with the sensor, a standalone and portable thermometer system.

Table 3 reports the main requirements of such a system. The operating frequency, above 10 GHz, poses some challenges in the design of the microwave front-end, which is in charge of down-converting the microwave signal into a low-frequency signal, suitable for direct analog-to-digital conversion. Single-step frequency conversion, which would be the simplest solution, becomes increasingly difficult at higher frequency. Moreover, all the necessary microwave components (attenuators, amplifier, filters, etc.) are increasingly expensive at higher frequency, thus the design must be carefully optimized to feature the desired low-cost requirements. The instrument dynamic range must be as large as possible to account for the high-Q nature of the resonance and for possible variability of transmission levels in different experiments or with temperature.

Last and most important, in order to achieve a potential sub-millikelvin temperature resolution, a resonance frequency relative measurement accuracy in the order of few parts in 10^9^ (parts-per-billion, ppb) is desirable, and a 1 ppb level stable frequency reference is required as an instrument synchronization time base.

**Figure 3:**
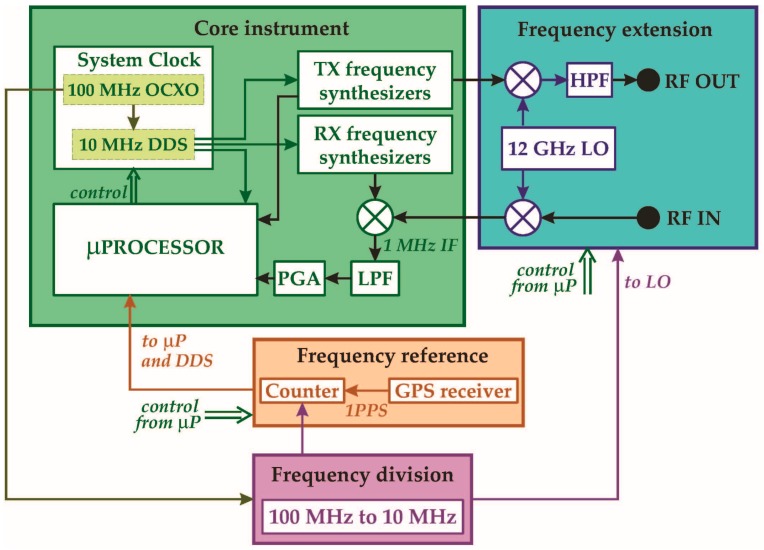
Whispering gallery mode thermometer (WGMT) acquisition system.

Figure 3 shows the block diagram of the ad hoc acquisition system designed for the selected WGM sensor. Four functional blocks can be identified: the first one is the instrument’s core (green), consisting of a “low-frequency” (800 MHz to 7 GHz) vector S_21_-meter, previously designed and assembled by the authors [14,15]. The second one is a single-step-conversion frequency extension module (blue), that enables the system to cover the 13–19 GHz bandwidth, while the third is a low-cost, high-accuracy, frequency reference module (red) specifically designed to match the WGMT requirements [12,13]. The fourth block is a simple frequency divider module (purple) generating a 10 MHz signal, which is required by the frequency reference and the frequency extension modules.

The most peculiar feature of the core instrument is the adoption of a finely tuned 10 MHz reference clock, thanks to which a resolution of 1 part in 10^8^ for the generated microwave signal is achieved, even employing low-cost, large-frequency-step microwave synthesizers. This special clock, generated by a direct digital synthesizer (DDS), is applied to all the core functional blocks, including the microprocessor, in order to maintain coherency and improve the signal-to-noise ratio. In addition, this approach enables the use of a simplified three-parameter sine-fit algorithm with coherent subsampling [16].

The frequency extension module performs a single-step frequency up-/down-conversion from the core instrument’s bandwidth to the 12.8–19 GHz range and vice versa, employing only one single 12 GHz local oscillator. A 12–20 GHz band-pass filter suppresses the low-band product frequencies, to avoid superposition with cavity modes.

The frequency reference directly affects measurement uncertainty. In order to make its contribution negligible with respect to the sensor’s intrinsic uncertainty, its accuracy must not exceed 1 part in 10^9^, a value that is typically achieved only by expensive laboratory frequency standards (e.g., rubidium clocks). The designed module, instead, adopts a GPS receiver to gather the 1-pulse-per-second (1 PPS) timing signal, whose accuracy and stability are guaranteed by the GPS satellite on-board atomic clocks, and uses it to accurately measure the local clock. The result is then used both to adjust the DDS and to correct the residual frequency error of each measured resonance. This solution requires no calibration, is extremely low-cost, and introduces no clock perturbations, avoiding direct feedback. The only additional requirement posed by this approach is the availability of a GPS signal in the operation site.

A post-processing software to implement Lorentzian fitting has been developed. The algorithm is based on the Levenberg–Marquardt method, optimized for complex resonances, and it is able to model up to three degenerated resonances. Transmission background is modeled with a fourth-order polynomial. Moreover, phase rotations introduced by the connecting cables are accounted for by a dedicated subroutine that numerically corrects raw measurements.

This software is embedded in a graphical desktop application that provides an interface to set up and control the instrument and manage the gathered raw data. This application also plots the real-time trend of the resonance frequency (or the mean of the resonance frequencies) during a measurement run.

## 3. Results

### 3.1. Thermometer System Validation

#### 3.1.1. Sensor Validation [9,10,11]

The initial experiments, aiming at characterizing the two WGM resonators, were carried out in a temperature range from −40 °C to +85 °C. The experimental setup included a Fluke calibration bath 7381, an ITS-90 calibrated standard PRT (SPRT), a Fluke 1560 resistance bridge, and a thermometric fixed point (ice point). An Agilent E5071C Network Analyzer and an SRS FS725 rubidium frequency standard were used to measure the resonant frequency, together with a simple Lorentzian fitting post-processing algorithm. The results of these preliminary experiments are summarized in Table 4.

#### 3.1.2. Acquisition System Validation [12,13]

Several experiments were carried out to validate the designed and in-house-assembled acquisition system [12,13]. The validation of the frequency reference accuracy was performed employing an ultrastable (0.1 mK/day drift) experimental setup from LNE-CNAM [17]. The available resonator was a 5.77 GHz quasi-spherical resonator (QSR), which did not require the frequency extension module. This was then validated in a different experiment as described below. The QSR has been monitored for 15 h with the developed instrument, gathering one spectrum every 74 s. All the measured frequency values were within 3 parts in 10^9^ (peak-to-peak) with a standard deviation of only 1.2 parts in 10^9^. A second 15 h measurement session was then performed with a state-of-the-art commercial VNA: the discrepancy between the mean frequency values obtained with the two instruments was only 10 Hz (i.e., 1.7 parts in 10^9^).

Since the extension module employs the same clock as the core instrument, we can infer that the results in the 13–19 GHz band have the same frequency accuracy as those obtained at 5.77 GHz, provided that the extension module does not introduce any nonlinearity.

For linearity and repeatability assessment, the WGM spherical resonator described in the previous section was monitored. Different modes at difference frequencies have been measured, including WGM m=3, WGM m=4 (weaker but measurable), and three cavity resonant modes. Linearity was verified by comparing the normalized residuals of the Lorentzian fit of resonance spectra obtained at different frequencies in the two different experiments: comparison of fitting residuals in the two instrument’s bands proves that the instrument response is linear in the entire frequency range. Repeatability was evaluated from consecutive measurements of the different resonances. The results, summarized in Table 5, demonstrate that the developed acquisition system can effectively replace a laboratory VNA-based acquisition setup.

### 3.2. Stability and Resolution of the Thermometer System

**Figure 4:**
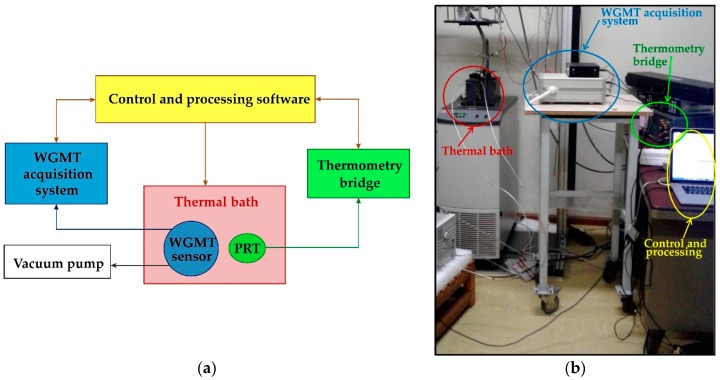
Setup for the investigation of temperature stability and resolution: (**a**) block diagram; (**b**) picture of the experimental system.

The stability of the whispering gallery mode thermometer system was carefully verified through a 48 h monitoring of the temperature of a calibration thermal bath (Fluke 7381) set around 20 °C. A calibrated Fluke 5620 PRT with 5 mK uncertainty (k=2) read by a Fluke 1590 thermometry bridge was used as reference. Figure 4 depicts the experimental setup: both temperature-sensing elements were placed inside the bath at the same depth in a region where the estimated maximum temperature gradient was below 4 mK. During all experiments, the WGMT was connected to a vacuum pump to minimize the cavity perturbation and maintain the sapphire surface clean and unperturbed.

**Figure 5:**
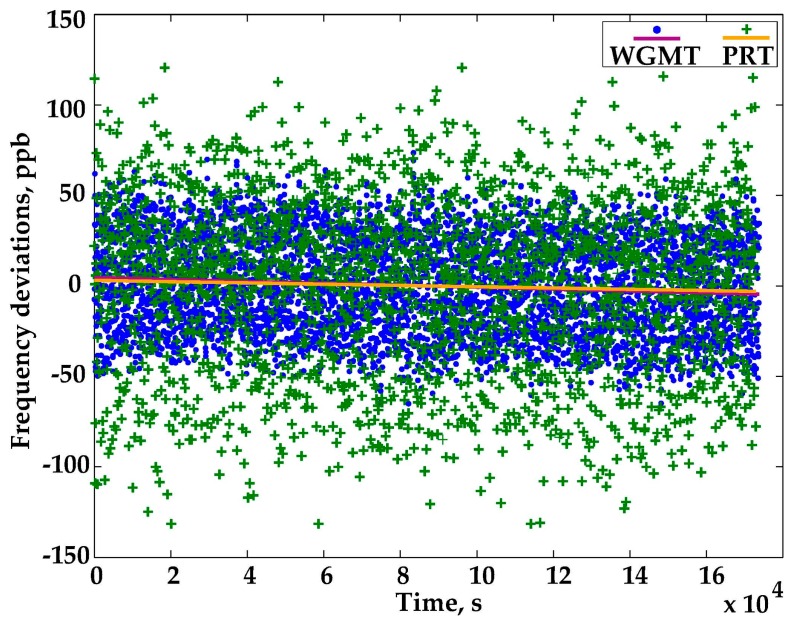
Long-term drift and temperature resolution at 20 °C: WGMT vs. PRT (platinum resistance thermometer). The measurement standard deviation of WGM is 3 × 10^−8^.

The results of the experiment are plotted in Figure 5: the frequency deviations recorded by the WGMT are compared with the temperature deviations observed by the PRT. The two quantities are made consistent by multiplying the PRT readings by the average sensitivity of the WGTM, which is −60 ppb/mK at 20 °C. The temperature drift observed by the two thermometers in 48 h, represented by the straight lines of the fit in Figure 5, is almost the same: −50 μK/day according to the PRT and −77 μK/day according to the WGMT. Such a small difference (below 30 μK/day) demonstrated the good stability of the WGMT system, while the plot’s scatter proved its high resolution, at least twice that of the PRT and thus able to detect temperature changes as low as few tens of microkelvins.

### 3.3. Thermometer Calibration Results

#### 3.3.1. Full-Range Calibration from −74 °C to 85 °C

The experimental setup adopted for the WGMT calibration was the same of that reported in Figure 4. For measuring temperatures below 0 °C, an ethanol bath was used; above 0 °C, the bath was filled with deionized water. A dedicated control software was in charge of collecting both the WGMT and PRT responses and changing the bath temperature every 6 h; the last 4 h of data acquisition were processed to extract the temperature values. In this time interval, the bath exhibited a temperature stability from ±0.5 mK to ±2 mK. The calibration curve was computed from the gathered PRT temperatures and WGMT resonance frequencies by fitting the data with a cubic polynomial of the form

(2) $$f_{0}=a_{0}+a_{1}T+a_{2}T^{2}+a_{3}T^{3}$$

The cubic approximation was adopted for all the experiments since adopting a fourth-order polynomial, as done in the first sensor validation experiments [9,10,11], brought no improvement in the fit residuals.

##### Measurement Set 1: −74 °C to 0 °C Range

**Figure 6:**
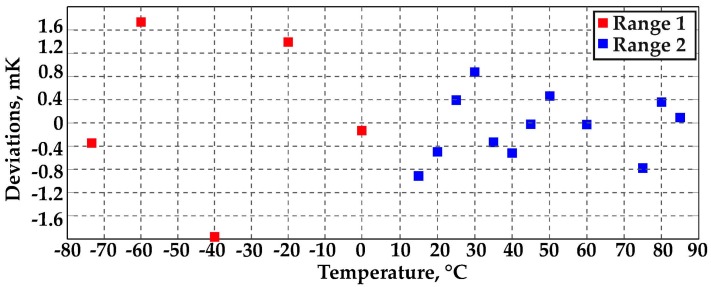
Calibration fit residuals: range 1 is −74 °C to 0 °C; range 2 is 10–85 °C (only the temperature points used for calibration curve fitting are shown, see [12]).

This temperature range was explored first: bath temperature was stabilized at 0 °C and then lowered down to −80 °C with 20 °C steps (actually the lowest temperature tested is −74 °C). At all investigated temperatures, the relative standard deviation of a single frequency measurement was below 1 × 10^−8^, which corresponds to less than 0.2 mK (with the exception of the point at −40 °C where the bath showed larger instability, thus the measurement standard deviation was around 0.7 mK). As shown in Figure 6 (red squares), the residuals of the cubic calibration curve were within ±2 mK peak-to-peak, which is a quite satisfying result considering the expected mechanical instability at low temperature (e.g., leak of sealing of the employed O-rings and differential thermal expansion between the cavity and the crystal support screw).

##### Measurement Set 2: 10–85 °C Range [12]

For temperatures above 0 °C, a 5 °C step was adopted, from 10 °C up to 85 °C. At all investigated temperatures, the relative standard deviation of a single frequency measurement was below 3 × 10^−8^. The worst-case relative standard deviation of the mean, considering ~200 acquisitions per measurement point, was about 2 × 10^−9^, which corresponds to a temperature standard deviation of less than 50 μK, confirming the very high resolution achievable by such a measuring system. As shown in Figure 6 (blue squares), the calibration curve fit residuals were within ±1 mK, a better result than that obtained in the negative temperature range.

##### Uncertainty Analysis

The combined uncertainty for a single measurement point for such experiments must account for the contributions of both the WGM thermometer system and the temperature calibration setup. The dominant WGMT uncertainty contributions were:

- frequency reference accuracy,
- Lorentzian fitting repeatability,
- short-term sensor repeatability, and
- calibration curve fitting,

giving a combined measurement uncertainty (k=1) of only 0.8 mK in the positive range and 1.3 mK in the negative range.

The dominant calibration setup contributions were:

- PRT calibration,
- bridge calibration, and
- bath temperature stability and uniformity,

giving a combined measurement uncertainty (k=1) of 5 mK.

The overall combined uncertainty, around 5 mK, can be almost fully ascribed to the employed calibration setup, which would be much improved by adopting a more accurate PRT (e.g., an ITS-90 standard PRT) and ensuring a lower temperature gradient between the sensors—for example, by adopting a capsule SPRT embedded within the WGMT cavity.

#### 3.3.2. Investigation of Narrow-Range Performance

Wide-range calibration accuracy proved to be largely limited by the mechanical stability of the WGM resonator inside the cavity and by the cavity sealing. A deeper investigation of this aspect is currently in progress, since, at present, it represents the main limiting factor on the achievable WGMT performance. In order to show the actual potential of the proposed WGMT, two further experiments were carried out with the following features:

- improved cavity sealing, by means of through screws along the cavity, and
- narrow temperature ranges and small temperature steps (1 K) to avoid stressing conditions for the WGMT assembly.

**Figure 7:**
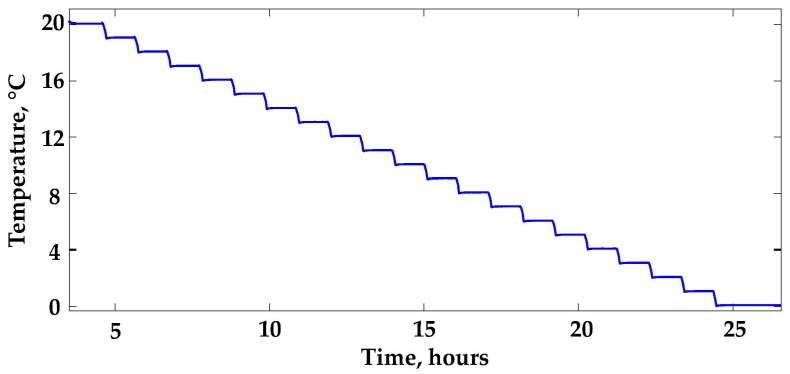
Temperature step profile.

Two temperature ranges were investigated: the range from 0–20 °C was explored first, then the wider range from −20 °C to 20 °C was considered. Fast acquisition intervals were adopted, as shown in Figure 7, reporting the temperature steps for the first experiment. The bath temperature was changed by 1 °C every hour, and the last 30 min of each acquisition were used to evaluate the temperature. In both tests, the bath was filled with ethanol.

**Figure 8:**
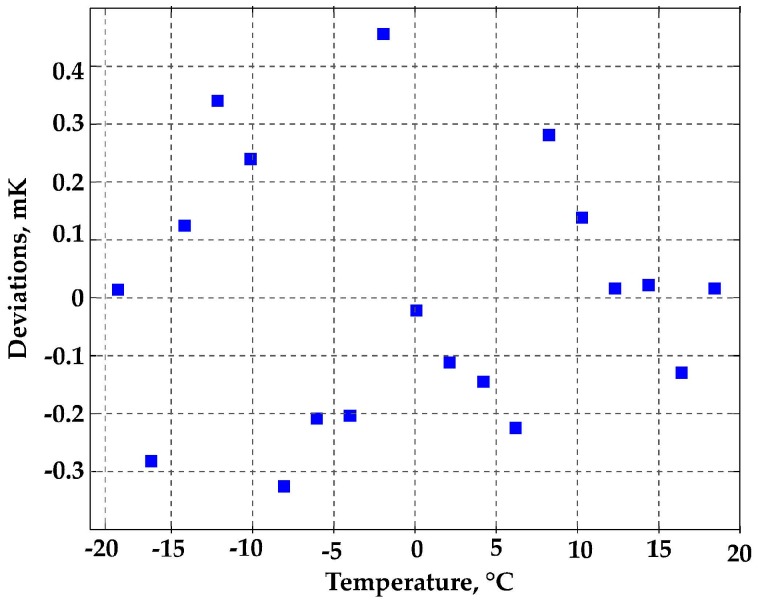
Calibration fit residuals: −20 °C to 20 °C range.

**Figure 9:**
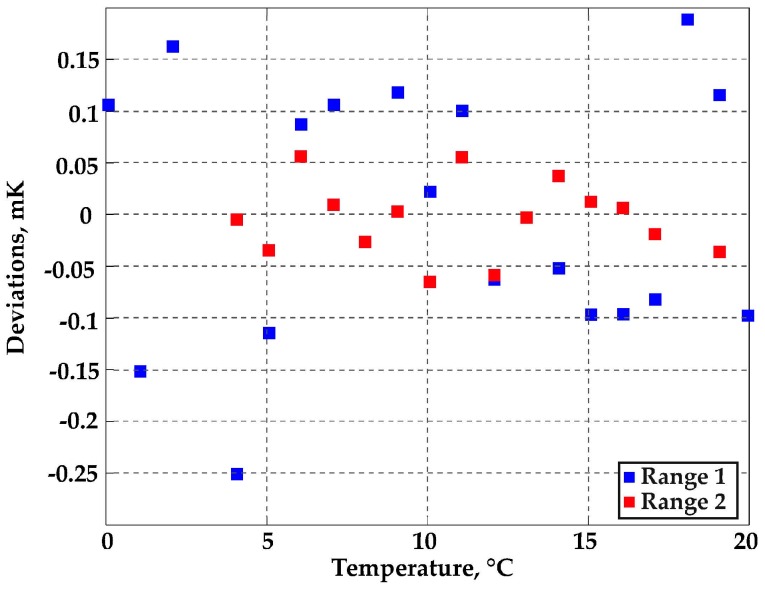
Calibration fit residuals: range 1 is 0–20 °C; range 2 is 4–19 °C.

Calibration fit residuals, as reported in Figure 8 and Figure 9, were within ±0.4 mK in the whole −20 °C to 20 °C range (Figure 8), and within ±0.25 mK in the range 0–20 °C (Figure 9, blue squares). In Figure 9, the results obtained in the subrange 4–19 °C are also reported (red squares), showing fit residuals within ±60 μK. These results demonstrated that the WGMT performance potentially achievable by improving the mechanical stability could be an order of magnitude better than that obtained in the full-range experiments and comparable with the accuracy of the best ITS-90 standard PRTs.

## 4. Discussion and Conclusions

Whispering gallery mode (WGM) sensors based on dielectric resonators have attracted a significant interest in recent years since they can afford very high sensitivity. On the other hand, for accurate metrology applications, besides measurement sensitivity itself, other key parameters are of interest, such as repeatability and reproducibility of the sensor system (i.e., the overall performance accounting for the means used for detecting the resonances and for converting the measured frequencies into the measurand of interest).

The results presented in this work indicate that the sapphire-based WGM approach, and its practical implementation, can provide a highly sensitive and accurate system for measuring the temperature in a wide range of operation. Here, it was also shown that the sensing principle is largely immune to the presence of spurious resonant modes near the selected resonance frequency. The reproducibility was mainly limited by the mechanical features, but insensitive to cavity size and polishing.

The experiments showed that below 0 °C (from −74 °C upwards), a relative standard deviation of a single frequency measurement better than one part in 10^8^ could be achieved. It corresponds to a temperature standard deviation lower than 200 μK, which is much better than the corresponding performance of the reference PRT adopted in the tests. On the other hand, above 0 °C and up to 85 °C the relative standard deviation of a single frequency measurement was always below 3 × 10^−8^. The worst-case relative standard deviation of the mean, by averaging over 200 acquisitions per measurement point, is about 2 × 10^−9^, which corresponds to a temperature standard deviation of the mean better than 50 μK. Repeated tests also showed a thermometer standard uncertainty associated with the short-term stability better than 150 μK at the ice melting point (as reported in [9], Section VI-B).

Although initially the aim of the studies here presented was to identify a potential candidate for PRT replacement in industrial applications, the actual WGM thermometer system capability outperformed standard industrial PRT characteristics (e.g., the calibration curve fit residuals are within 1 mK above 0 °C), prompting for its use in higher accuracy research applications as well. In particular, the extremely good results obtained in the narrow temperature range from 4–19 °C, exhibiting fit residuals within 60 μK, showed the potential of the proposed WGM thermometer system as an interpolating thermometer for the dissemination of the thermodynamic temperature scale, and as a powerful tool for investigating the resistivity vs temperature characteristics of the present ITS-90 interpolating SPRTs.

## Figures and Tables

**Table 1:** Simulation results of the cylindrical WGM resonator.

Mode m	$f_{0}$, GHz	Q
3	12.405	3.0 × 10^5^
4	15.147	1.5 × 10^6^
5	17.841	4.7 × 10^6^

**Table 2:** Simulation results of the spherical WGM resonator.

Mode m	$f_{0}$, GHz	Q
3	13.536	3.0 × 10^5^
4	16.345	2.0 × 10^6^
5	19.068	6.1 × 10^6^

**Table 3:** Acquisition system requirements.

Dynamic range	>60 dB
Operating frequency	>10 GHz
Frequency measurement rel. uncertainty	<10^−8^
Frequency reference rel. accuracy	<10^−8^
Frequency reference rel. stability	<10^−9^

**Table 4:** Sensor testing results [9,10,11].

	Cylindrical Resonator	Spherical Resonator
Q (worst case at −40 °C)	1.7 × 10^5^	1 × 10^5^
Fractional frequency sensitivity at −40 °C	−56 ppb/mK	−56 ppb/mK
Fractional frequency sensitivity at +85 °C	−67 ppb/mK	−67 ppb/mK
Ice melting point repeatability (peak-to-peak)	±0.4 mK	±0.5 mK
Ice melting point stability (peak-to-peak)	±2 mK	±0.5 mK
Sensor interchangeability	-	±40 mK
Sensor reproducibility	±20 mK	-

**Table 5:** Acquisition system’s validation results.

$f_{0}$, GHz	Mode	Fit Residuals *	Standard Deviation
5.77	QSR	6.7 × 10^−4^	1.2 × 10^−9^
13.6	WGM m=3	4.2 × 10^−4^	2.3 × 10^−9^
14.8	Cavity	3.8 × 10^−4^	3.8 × 10^−9^
16.4	WGM m=4	6.1 × 10^−4^	4.3 × 10^−9^
17	Cavity	4.3 × 10^−4^	4.4 × 10^−9^
17.7	Cavity	4.9 × 10^−4^	3.7 × 10^−9^

* Maximum value of the normalized Lorentzian fit residuals.
